# Hydroethanolic *Extract of A. officinarum* Hance Ameliorates Hypertension and Causes Diuresis in Obesogenic Feed-Fed Rat Model

**DOI:** 10.3389/fphar.2021.670433

**Published:** 2021-07-09

**Authors:** Farah Javaid, Malik Hassan Mehmood, Bushra Shaukat

**Affiliations:** Department of Pharmacology, Faculty of Pharmaceutical Sciences, Government College University of Faisalabad, Faisalabad, Pakistan

**Keywords:** *Alpinia officinarum*, diuretic, leptin, adeponectin, obesity induced hypertension

## Abstract

*Alpinia officinarum* Hance (*Zingiberaceae*) has been used widely in traditional Chinese and Ayurvedic medicines. Its folkloric uses include relieving stomach ache, treating cold, improving the circulatory system, and reducing swelling. Its effectiveness and mechanism of antihypertension in obesity-induced hypertensive rats have not been studied yet as per our knowledge. This study has been designed to provide evidence of underlying mechanisms to the medicinal use of *A. officinarum* as a cardiotonic using an obesity-induced hypertension model in rats. Chronic administration of *A. officinarum* caused a marked reduction in the body weight gain and Lee index of rats compared to the obesogenic diet-fed rats. Its administration also caused attenuation in blood pressure (systolic, diastolic, and mean), serum total cholesterol, triglyceride, and leptin, while an increase in serum HDL and adiponectin levels was noticed. The catalase and superoxide dismutase enzymatic activities were found to be remarkable in the serum of *A. officinarum*-treated animal groups. *A. officinarum* showed mild to moderate diuretic, hepatoprotective, and reno-protective effects. *The A. officinarum-*treated group showed less mRNA expression of 3-hydroxy-3-methylglutaryl-CoA reductase while the mRNA expression of peroxisome proliferator-activated receptor and mRNA expression of cholesterol 7 alpha-hydroxylase were raised in comparison to the hypertensive group of rats evaluated by quantitative real-time polymerase chain reaction. These findings show that *A. officinarum* possesses antihypertensive and diuretic activities, thus providing a rationale to the medicinal use of *A. officinarum* in cardiovascular ailments.

## Introduction

Obesity is a chronic metabolism-related disorder and it is correlated with various pathological conditions including hypertension, atherosclerosis, cardiovascular disease, hyperlipidemia, and diabetes mellitus (Férézou-Viala et al., 2007). In a community irrespective of gender differences, there are equal rising rates of hypertension which are strongly associated with the growing number of obese people in the population (Faulkner and Belin de Chantemèle, 2018). The exact pathway of obesity-induced hypertension is still unclear. However, many studies have revealed that derivatives of adipose tissues, metabolic system, and modulation of presser and depressor are involved. Obesity-induced hypertension is thought to be the result of intermixing of the aforementioned factors (Kotsis et al., 2010). Ingestion of a high amount of sugar and fat may also be linked with an enhanced amount of reactive species which in turn impairs the endothelium. The resultant oxidative stress is known to develop arterial high blood pressure (Parik et al., 1996; Roberts et al., 2000; Sharma et al., 2007). While, the best-known mechanisms for salt intake-associated hypertension models after 7 days are altered hemodynamics, vascular injury, impaired Na^+^ homeostasis, and inflammation (Yan et al., 2019). A survey from the Framingham Heart Study reported that obesity is the interlinking of multifactorial morbidities which is highly associated with essential hypertension (Wilson et al., 2002). Chronic obesity leads to an increase in blood pressure then it becomes difficult to control by mere intervention of antihypertensive drugs (Modan et al., 1991). However, a decrease in body weight is more important in the prevention and treatment of obesity-related hypertension (Neter et al., 2003).

Traditional medicinal plants are used in major developing countries for the maintenance of better health. In Europe and China, the *Alpinia officinarum (AO)* rhizome has been used traditionally for the improvement of the circulatory system, relieving diabetes, stomach ache, swelling, and colds (Basri et al., 2017). The rhizome of AO has been reported for its anti-obesity, hypolipidemic, anticoagulation, anti-oxidant, antidiabetic, anti-ulcer, antidiarrheal, anti-emetic, analgesic, anti-inflammatory, anticancer, antipsychotic, and antibacterial activities in various studies (Abubakar et al., 2018). Among various biologically active phytochemicals, flavonoids and natural phenolic compounds act as powerful antioxidants in the body. Fruits and vegetables that have a high amount of flavonoids and can prevent the development of cardiovascular disease (Horáková, 2011). Polyphenolic compounds such as catechins have many physiological roles in the body, particularly protecting the cardiovascular system from various diseases (Chen et al., 2016). Kaempferol is majorly obtained from fruits, vegetables, and tea that may have cardioprotective action (Dabeek and Marra, 2019). The mixture of the high amounts of fat, sucrose, and salt in a diet has been used to mimic obesity-induced hypertension in a rat model (Dobrian et al., 2003; Sharma et al., 2007). AO has activity against obesity and hyperlipidemia (Xia et al., 2010) but to date, no scientific validation is available for the effect of AO on obesity-induced hypertension. The purpose of this study was to provide scientific evidence to back up the vernacular claim that AO invigorates the circulatory system using obesity-induced hypertension and diuresis in rats due to its active constituents such as phenolic compounds and flavonoids.

### Novelty

• It is the pioneer study highlighting the antihypertensive effect of *Alpinia officinarum in obesity-induced hypertension.*
• An innovative diet model was developed containing high fat (beef tallow), salt and sugar. Such a diet has been used for the first time to develop obesity-induced hypertension.
• *A. officinarum* showed its diuretic potential which could be considered as an add-on effect contributing to its antihypertensive effect.
• The higher the dose, the higher the response was observed.

## Material and Method

### Chemicals

The crude extract of *Alpinia officinarum* Hance (Zingiberaceae) (AO) was provided by Salus Company, China. Analytic reports for batch number 180720 (*A. officinarum*) are available for the extract. The analytic reports, as well as a portion of the extract used, were as stipulated, stored at the Department of Pharmacology, Faculty of Pharmaceutical Sciences, Government College University, Faisalabad, Pakistan. Other ingredients including powdered milk (Nido, Nestle Pakistan Limited, Lahore, Pak), Table salt, vegetable oil (Sufi oil, Lahore, Pakistan), and nutrivet-L were obtained from the commercial supplier. While chokar, wheat flour, fishmeal, molasses, 10% formalin, and potassium metabisulfite were purchased from the local market. Atorvastatin (Brand Aotrva, Pharmatec, Pakistan) and Furosemide (Lasix, Sanofi Aventis, Karachi, Pakistan) were obtained from the local pharmacy.

### Animals

All experiments of the study were performed on rats obtained from the Faculty of Pharmaceutical Sciences, Government College University, Faisalabad (GCUF). This study is a portion of the main project, submitted to comply with the requirements for the degree of Doctor of Philosophy in Pharmacology. This study was approved by the Board of Advanced Studies and Research, GCUF. The study was performed on female Wistar albino rats (155–220 g). They were accommodated at the animal house at GCUF where the surrounding temperature was controlled to be between 23 and 25°C. Water and food were made freely available to all the animals. Animal ethical protocols were followed as per the institutional guidelines and approved by the institutional review board (IRB 761).

### High-Performance Liquid Chromatographic Evaluation

A high-performance liquid chromatographic (HPLC) system (Shimadzu, Japan) was provided with the LC 10AT pump, bipolar column heater, and gradient detector (UV-Visible, SPD-10AV). Separation was done on the C18 guard column and the mobile phase used was methanol: water: phosphoric acid (60:38:2, v/v/v, isocratically) (Tao et al., 2006). The rate of flow was constantly maintained at 0.8 ml per minute and the peaks that appeared were recognized with the help of UV at 280 nm absorbance. The column temperature was kept at 40°C during analysis.

A UV-visible-type detector was used for the segregation of flavonoids and phenolic compounds. The identification of these flavonoids and phenolic compounds was done by comparing the retention time and UV-visible spectra of the peaks, with the spectral peaks previously obtained by the injection of standards. The calculation for their quantification was performed by external calibration.

### Composition of Animal Diets

Two different types of diets were used.

#### Standard Diet (SD)

The standard diet was prepared at the GCUF. It was comprised of 2 kg of dried powdered milk, 5 kg of chokar, 5 kg of wheat flour, 2.25 kg of fishmeal, 75 g of table salt, 150 g of molasses, 15 g of potassium metabisulfite, 500 g of vegetable oil, and 33 g of nutrivet-L. The solid ingredients were ground until powdered and then made into a smooth mixture that formed an amount of almost 15 kg. Some water was mixed into the powder mixture to make a soft mixture out of which a lump of 300 g was prepared (Harkness et al., 2010).

#### High Fat Sugar Salt (HFSS) Diet

An earlier protocol (Ragab et al., 2015) was followed with some amendments. A total of 55% of the standard diet was supplemented with 10% sugar, 2% salt, and 33% beef tallow (wt/wt). They were mixed with powdered components of SD until semisolid so that all parts were mixed equally in the diet.

### Hypertension Experimental Design

After the one week of acclimatization, the two main groups of rats were decided: one group contained normotensive rats (*n* = 6) fed on an SD and the other group (*n* = 30 rats) was fed the HFSS diet (SD 55%, beef tallow 30%, sucrose 10%, and NaCl 2%) for 6 weeks (induction period). The rats fed on the HFSS diet were screened at the end of 6 weeks for the development of hypertension and only 24 hypertensive rats were subdivided into the following four groups for the next 6 weeks as detailed below.

**Table:** 

Groups	Intervention
Normotensive group	Only SD
Hypertensive group	Only HFSS
Atorvastatin group	HFSS + atorvastatin (10 mg/kg/day in normal saline) PO (Liu et al., 2014)
AO low dose (AO 250 mg/kg) group	HFSS +250 mg/kg AO extract (orally)
AO high dose (AO 500 mg/kg) group	HFSS +500 mg/kg AO extract (orally)

All of these were orally administered SD, HFSS diet. and plant extracts every day for a further 6 weeks (treatment period).

### Noninvasive Blood Pressure Measurement (NIBP) Method

To assess the onset and development of hypertension, the NIBP (ML125, NI-0541, AD instrument, Sydney, Australia) apparatus was used by employing the tail-cuff method. During the use of NIBP, the temperature of rats was maintained at 35°C. The cuff sensor was set up on their tail while keeping the rat in a restrainer of appropriate size. The tail cuff was distended by pressing the start button, after then the cuff slowly released the pressure. The PowerLab data acquisition system with Lab Chart 7.0 software (Ad Instruments, Sydney, Australia) was used for recording pulses. Direct pulse tracing was done for interpretation of systolic blood pressure (SBP), mean blood pressure (MBP), and heart rate (HR). However, diastolic blood pressure (DBP) was estimated by the following equation:

DBP = (3MBP − SBP)/2 (Ayele et al., 2010).

Parameters of blood pressure were measured under a conscious state at 1, 3, 6, 8, 10 and 12 weeks into the diet. For each measurement, the average of three pressure readings was recorded.

### Body Weight, Body Length, Lee Index, and Weight of Different Body Organs

Individual body weight (g) and naso-anal length (cm) were recorded at 1, 3, 6, 8, 10 and 12 weeks into the diet. The Lee index (obesity index) was obtained after dividing the cube root of body weight (g) by naso-anal length (cm) and multiplying the result by 1,000. To estimate obesity, the Lee index in rats, described by Lee in 1929, is comparable to body mass index in humans (Lee, 1929). Since then many investigators have utilized it to estimate the level of obesity in rats. In a few studies, a reliable relation between adipose tissue and Lee index of the body was found (Li et al., 1997; Zhi et al., 2002).

### Biochemical Analysis

After 12 weeks of the experiment, the animals were starved for 16–18 h and euthanized by anesthesia with chloroform by inhalation in a closed chamber. The blood was obtained via cardiac puncture from every rat in the vacutainer. All vacutainers were placed in an upright position for 45 min at room temperature. The collected blood was then centrifuged at 400 × g for 5 min to obtain serum and stored at −80°C.

#### Determination of Lipid Profile Indices

The amount of total cholesterol (TC), total glycerides (TG), and high-density lipoprotein cholesterol (HDL-C) were obtained from serum samples. Standardized enzymatic procedures were followed using commercial kits (Germany). The low-density lipoprotein fraction of the cholesterol (LDL-C) was obtained by subtracting HDL-C from TC.

#### Estimation of Liver Profile Indices and Renal Profile Indices

Liver profile indices (alkaline phosphatase (ALP), alanine aminotransferase (ALT), aspartate aminotransferase (AST), total protein and globulin) and renal profile indices (creatinine and urea) were measured from isolated serum samples. Results were shown as IU/L and mg per deciliter of serum at the pathological laboratory of the University of Veterinary and Animal Sciences, Lahore.

#### Estimation of Leptin and Adiponectin in Serum

The serum biomarkers of obesity, leptin (E-EL-R0329), and adiponectin levels (E-EL-R0329) were measured through an ELISA kit (Elab science, United States) by following the manufacturer’s protocol. The reaction mixture was provided with serum (100 ul) in already coated wells and kept at 37^°^ in the ELISA plate reader (DIA source, Germany). The reaction was monitored at a wavelength of 450 nm. Serum leptin and adiponectin levels were expressed as ng/ml and pg/ml, respectively.

### Histopathological Analysis

The heart, kidney, and liver of rats were dissected and then placed in formalin (10%) for 3 days. The small parts of these organs were dehydrated and fixed in paraffin wax. A microtome (Leica, Germany) was used to collect thin sections of the left ventricle (5 mm), kidney (5 μm), and liver (5 μm). Finally, these sections were dyed with histological stains (hematoxylin and eosin) and examined under a light microscope.

### Antioxidant Study on Tissue Homogenate

The antioxidant activities were analyzed by measuring the amount of CAT and SOD on the tissue homogenate. All animals were anaesthetized and sacrificed by cervical dislocation. The organs (liver, kidney, heart, and aorta) were removed and washed with normal saline and stored at −80°C. The experiments were performed by following earlier practiced methods (Aebi, 1974; Kakkar et al., 1984).

### Estimation of Diuretic Activity

One week before the terminal day of the study, rats were kept in separate metabolic cages for acclimatization. At 14–16 h before starting the diuresis experiment, animals were not given food but had full access to drinking water. At the end of the model, urine samples were measured twice, at the 5th and 24th h after administration of the doses of plant extract. All urine samples were collected in the container, strained to separate the fecal contents, and preserved at −20°C to assess the excretion of electrolytes. All drugs were administered orally.

**Table:** 

Groups	Intervention
Normotensive rats	Normal saline only
N\S hypertensive group	Normal saline only
Furosemide hypertensive group	furosemide (10 mg/kg/body weight) orally (Hailu and Engidawork, 2014; de AF Da Silva et al., 2015)
AO (250 mg/kg)	250 mg/kg AO extract (orally)
AO (500 mg/kg)	500 mg/kg AO extract (orally)

#### Assessment of Urine Parameters

The total urine volume of all animals was measured at the 5th and 24th h after the last administration of the test dose of the extract. An electrical conductivity meter and a digital pH meter were used to measure urinary conductivity and pH, respectively, immediately after collecting urine. To assess the actual amount of urinary ions (as Na+, K+, and Cl^−^), the samples of total urine were mixed with demineralized water (1:1,000) (Hailu and Engidawork, 2014).

#### Assessment of Diuretic Action and Diuretic Activity

The ratio of urine output in the treated group and normotensive groups was considered as diuretic action. The diuretic activity was calculated from the ratio between the test group and hypertensive groups. Before the commencement of the experiment, it was decided that diuretic activity will be assumed good (>1.50), moderate (1.00–1.50), little (0.72–1.00), and nil (<0.72) (Hailu and Engidawork, 2014).

#### Assessment of Saluretic, Natriuretic, and Carbonic Anhydrase Inhibition

Saluretic activity was obtained by adding sodium ions and chloride ions excreted in the urine. The ratio of sodium and chloride ions was employed as natriuretic activity. The ratio between chloride ions and Na^+^ + K^+^ was used for the calculation of carbonic anhydrase inhibition (Somova et al., 2003).

### Quantitative Reverse Transcription Polymerase Chain Reaction (qRT-PCR)

Estimation of mRNA expression of peroxisome receiver activator alpha (*PPARα*), 3-hydroxy-3methyl-glutaryl-coenzyme A reductase (*HMGR*), 7α-hydroxylase (*CYP7A1*), and β-Actin was done by qRT-PCR. Frozen liver tissues were homogenized by using Trizol reagent (Invitrogen, Thermo Fisher Scientific) and total RNA was extracted by following protocols by Thermo Fisher Scientific. Approximately 2 µg of the total RNA from each sample was used for cDNA synthesis using a high capacity cDNA reverse transcriptase kit (Molecular Biology, Thermoscientific). The cDNA was subsequently amplified using Syber Green PCR (Molecular Biology, Thermoscientific). Primers’ stock solutions were used according to the supplier’s protocol (e-Oligos, Newyork, United States). PCR for *PPARα, HMGR, and CYP7A1* was performed in a final volume of 15 µL in the presence of 1 µL of cDNA as a template, 7.5 µL of Sybergreen, 5.9 µL of RNAase Free water, 0.3 µL of primer(f), and 0.3 µL of primer (r) into each of the following sets of primers. 5’-TCA​CAC​AAT​GCA​ATC​CGT​TT-3’ (sense) and 5’-GGC​CTT​GAC​CTT​GTT​CAT​GT-3’ (antisense) for *PPARα*, product size: 177 bp; 5’- TGC​TGC​TTT​GGC​TGT​ATG​TC -3’ (sense) and 5’- CCC​TTT​GGG​TTA​CTG​GGT​TT-3’ (antisense) for *HMGR*, product size: 187 bp; 5’-CAC​CAT​TCC​TGC​AAC​CTT​TT-3’ (sense) and 5′- GTA​CCG​GCA​GGT​CAT​TCA​GT-3’ (antisense) for *CYP7A1*, product size: 170 bp; and 5’- GTC​GTA​CCA​CTG​GCA​TTG​TG-3’ (sense) and 5’-CTC​TCA​GCT​GTG​GTG​GTG​AA-3’ (antisense) for β-Actin, product size: 181 bp. Annealing temperature for all primers was 60°C for 60−s. Cycle threshold (CT) values were normalized to the internal β-Actin control and ratios were detected using the CT method.

### Statistical Analysis

GraphPad Prism 7.0 software was employed for calculation and statistics. All values were presented as mean ± standard error of the mean (SEM). The values were statistically analyzed with the help of one-way analysis of variance (ANOVA) followed by Bonferroni test for bar charts and two-way analysis of variance (ANOVA) followed by Bonferroni test for multiple comparisons for tabulated data. Values with *p* < 0.05 were considered statistically significant.

## Results

### High-Performance Liquid Chromatographic (HPLC) Analysis

**FIGURE 1:**
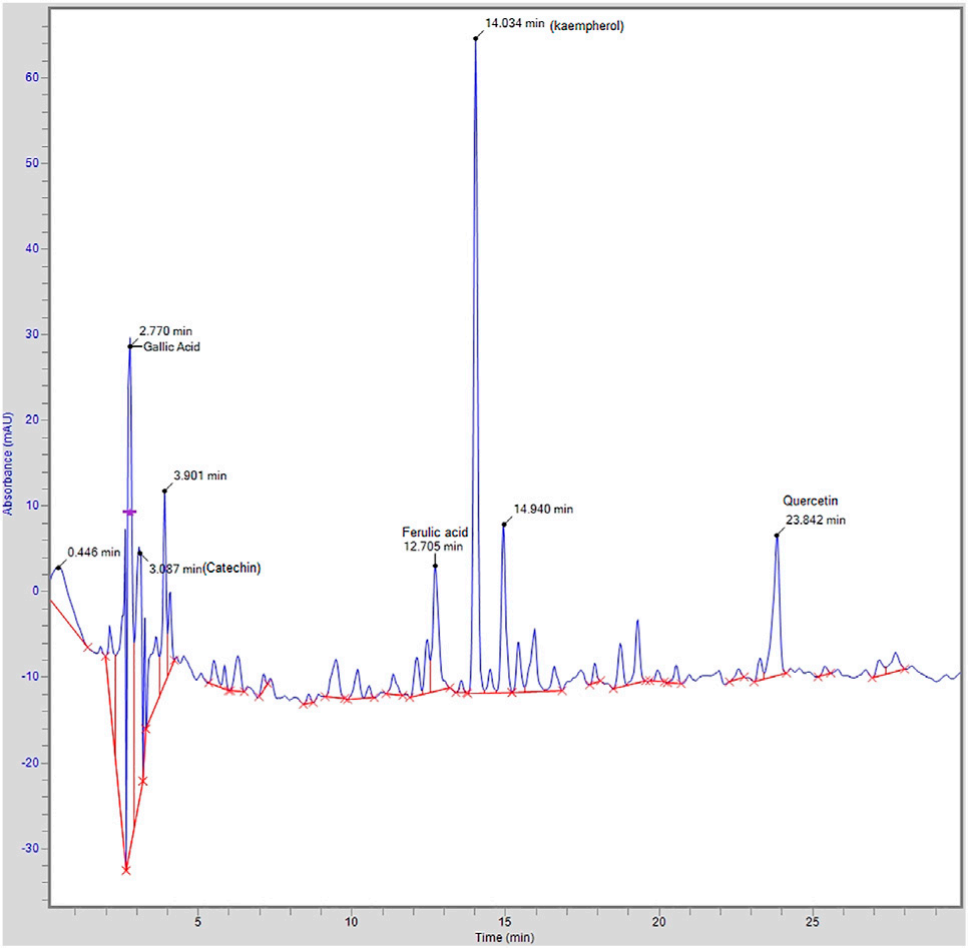
HPLC chromatogram of aqueous ethanolic extract of *A. officinarum.*

HPLC analysis of *A. officinarum* was performed for the assessment of compounds present in the hydroethanolic extract of the *A. officinarum* rhizome. The achieved chromatographs of the test material were compared with the standards. The identified compounds with retention time and area are shown in Figure 1. The HPLC chromatogram of the hydroethanolic extract of the *A. officinarum* rhizome found gallic acid (40 ppm), catechin (105.97 ppm), ferulic acid (10.37 ppm), kaempferol (194.71 ppm), and quercetin (26.64 ppm) present in the plant as active constituents (Figure 1).

### Effect of *A. officinarum* on Body Weight, Lee Index, and Weights of Different Body Organs

Weight gain (%) was calculated periodically for 12 weeks during the study. The animals of the hypertensive group were fed a HFSSD for an initial 6 weeks which caused a significant (*p* < 0.001) increase in weight gain and Lee index of rats compared to normotensive rats on SDs. Administration of AO (250 and 500 mg/kg) or atorvastatin (10 mg/kg) for the next 6 weeks significantly (*p* < 0.001) suppressed the rise in weight gain and Lee index when compared to hypertensive rats as seen in Table 1 and Table 2.

**TABLE 1:** Effect of chronic administration of *Alpinia officinarum* on weight gain (g) in obesogenic diet-fed hypertensive rats.

Groups	Induction period		Treatment period		
	Wk3	Wk6	Wk8	Wk10	Wk12
Normotensive	19.0 ± 1.5	32.0 ± 0.9^ɣ^	44.0 ± 2.3^ɣ^	59.0 ± 2.8 ^ɣ^	71.0 ± 1.4 ^ɣ^
Hypertensive	24.0 ± 1.4	52.0 ± 2.4^*c*^	85.5 ± 2.7^*c*^	101.5 ± 2.3^*c*^	134.5 ± 3.5 ^*c*^
Atorvastatin	25.5 ± 1.3	49.0 ± 1.4^*b*^	54.5 ± 2.0 ^ɣ^	63.5 ± 1.6^ɣ^	73.0 ± 2.4 ^ɣ^
AO (250 mg/kg)	23.0 ± 2.2	58.5 ± 2.4^*c*^	96.2 ± 3.9^*c*^	106.0 ± 3.6^*c*^	93.5 ± 4.9 ^*c*, ɣ^
AO (500 mg/kg)	25.8 ± 2.9	63.8 ± 1.8^*c*^	93.0 ± 3.5^*c*^	101.0 ± 3.8^*c*^	79.5 ± 1.9 ^ɣ^

Effect of chronic administration of *A. officinarum (AO)* on weight gain (g) in obesogenic diet-fed hypertensive rats. Normotensive group, Standard diet; Hypertensive group, High fat, sucrose and salt (HFSS) diet; Atorvastatin group, HFSS + Atorvastatin (10 mg/kg/day in normal saline, per oral); AO (250 mg/kg, per oral) group, HFSS + AO (250 mg/kg, per oral); AO (500mg/ kg) group, HFSS + AO (500mg/ g). The results are stated as means ± S.E.M (*n* = 6), where ^a=^ *p* < 0.05, ^b=^ *p* < 0.01, and ^c=^ *p* < 0.001 vs normotensive rats. ^α=^ *p* < 0.05, ^β=^ *p* < 0.01, and ^ɣ=^ *p* < 0.001 vs hypertensive rats.

**TABLE 2:** Effect of administration of *Alpinia officinarum (AO)* on Lee index in chronic obesogenic diet-fed hypertensive rats.

Groups	Induction period			Treatment period		
	Wk1	Wk3	Wk6	Wk8	Wk10	Wk12
Normotensive	299.4 ± 1.6	295.5 ± 1.1^β^	294.0 ± 2.8^ɣ^	291.6 ± 3.2^ɣ^	293.3 ± 3.0^ɣ^	291.5 ± 1.3^ɣ^
Hypertensive	293.8 ± 3.1	307.2 ± 1.2^*b*^	316.3 ± 1.8^*c*^	332.2 ± 2.1^*c*^	333.3 ± 3.0^*c*^	337.7 ± 2.9^*c*^
Atorvastatin	301.3 ± 0.8	312.8 ± 2.9	330.9 ± 3.4^*c*^	317.9 ± 3.2^*c*,*^	311.9 ± 1.9^*c*, ɣ^	294.5 ± 2.2^ɣ^
AO (250 mg/kg)	290.6 ± 3.7^a^	299.1 ± 2.9	319.6 ± 2.0^*c*^	329.4 ± 4.9^*c*^	324.0 ± 5.0^*c*, α^	293.0 ± 5.0^ɣ^
AO (500 mg/kg)	292.5 ± 3.7	306.5 ± 4.8^*b*^	323.3 ± 3.1^*c*^	321.9 ± 3.4^*c*, β^	307.9 ± 3.3^*c*, ɣ^	289.8 ± 2.4^ɣ^

The results are stated as means ± S.E.M (*n* = 6). ^a=^ *p* < 0.05, ^b=^ *p* < 0.01, and ^c=^ *p* < 0.001 vs normotensive rats. ^α=^ *p* < 0.05, ^β=^ *p* < 0.01, and ^ɣ=^ *p* < 0.001 vs hypertensive rats.

After 6 weeks of treatment, no significant change was seen in organ weight (left ventricle, heart/body weight, kidney) among animals of the treatment groups (AO, 250 and 500 mg/kg and atorvastatin) when compared to their respective normotensive and hypertensive rats. Concerning the weight of the heart and liver, a marked decrease (*p* < 0.001) was noticed in all treated groups compared to hypertensive rats (Table 3).

**TABLE 3:** Effect of administration of *Alpinia officinarum* on weight of various organs in chronic obesogenic diet-fed hypertensive rats.

Groups	Heart (mg)	Left ventricle (LV) (mg)	Heart/BW (mg/g)×100	Kidney (mg)	Liver (mg)
Normotensive	900.0 ± 36.5^ɣ,α^	196.0 ± 1.1	497.8 ± 37.4	850.0 ± 42.8	7,750 ± 381.9^ɣ^
Hypertensive	1,480 ± 206.2^*a*^	480.0 ± 19.8	616.7 ± 78.7	1,060 ± 32.4	9,650 ± 214.1^*c*^
Atorvastatin	975.0 ± 21.4^α^	245.0 ± 4.2	527.4 ± 12.3	880.0 ± 27.8	8,150 ± 293.0^ɣ^
AO (250 mg/kg)	930.0 ± 17.1^α^	270 ± 8.5	481.19 ± 19.1	930 ± 22.7	8,375 ± 128.3^*b*, ɣ^
AO (500 mg/kg)	900.0 ± 4.5^α^	248.3 ± 5.5	494.7 ± 7.1	840.0 ± 23.1	8,156 ± 180.2^ɣ^

Effect of administration of *A. officinarum (AO)* on weight of various organs in chronic obesogenic diet-fed hypertensive rats. The results are stated as means ± S.E.M (n = 6). ^a=^ *p* < 0.05, ^b=^ *p* < 0.01, and ^c=^ *p* < 0.001 vs normotensive rats. ^α=^ *p* < 0.05, ^β=^ *p* < 0.01, and ^ɣ=^ *p* < 0.001 vs hypertensive rats.

### Hypotensive Effect of the Extract of *A. officinarum*

Systolic blood pressure (SBP), diastolic blood pressure (DBP), mean blood pressure (MBP), and heart rate (HR) were assessed over the entire period of study. During the induction period, a noticeable rise (*p* < 0.001) in the SBP, DBP, MBP, and HR in all groups was observed compared to normotensive rats. After 6 weeks of treatment, relative to hypertensive rats, all treatment groups showed a significant decrease (*p* < 0.001) in SBP, DBP, MBP, and HR as detailed in Table 4 and Table 5

**TABLE 4:** Effect of administration of *Alpinia officinarum* on systolic blood pressure, diastolic blood pressure, and mean blood pressure in obesogenic diet-fed hypertensive rats.

Parameters	Duration		Groups				
			Normotensive	Hypertensive	Atorvastatin	AO (250 mg/kg)	AO (500 mg/kg)
SBP (mmHg)	Induction period	Week 1	122.9 ± 1.1	124.3 ± 1.1	119.3 ± 0.9	120.8 ± 1.8	126.8 ± 1.1
		Week 3	124.0 ± 0.8^β^	133.2 ± 2.0^*b*^	130.7 ± 0.6	128.3 ± 1.1	141.4 ± 1.1^*c*,α^
		Week 6	129.2 ± 0.7^ɣ^	142.9 ± 3.4^*c*^	138.9 ± 1.9^*b*^	138.2 ± 0.8^*b*^	141.4 ± 2.3^*c*^
	Treatment period	Week 8	125.9 ± 1.1^ɣ^	146.9 ± 4.6^*c*^	136.9 ± 1.9^*c*, β^	145.3 ± 1.5^*c*^	138.6 ± 2.0^c, α^
		Week 10	125.4 ± 1.2^ɣ^	150.6 ± 2.5^*c*^	130.6 ± 1.3^ɣ^	138.4 ± 2.3^*c*, ɣ^	132.5 ± 1.5^ɣ^
		Week 12	125.4 ± 1.2^ɣ^	154.4 ± 1.5^*c*^	126.7 ± 2.1 ^ɣ^	133.8 ± 1.7^*c,*^ ^ɣ^	126.5 ± 2.2 ^ɣ^
MBP (mmHg)	Induction period	Week 1	94.8 ± 3.9	88.4 ± 3.6	88.2 ± 2.7	92.2 ± 2.6	94.4 ± 3.7
		Week 3	92.4 ± 3.8	98.8 ± 2.7	104.1 ± 4.5	93.1 ± 2.4	107.0 ± 4.2^*a*^
		Week 6	96.0 ± 3.7^ɣ^	115.3 ± 2.5^*c*^	113.5 ± 1.8^*b*^	112.2 ± 2.3^*b*^	107.7 ± 2.3
	Treatment period	Week 8	94.1 ± 1.9^ɣ^	122.8 ± 4.9^*c*^	110.8 ± 5.8^*b*^	109.5 ± 4.5^*a*^	105.5 ± 3.0^β^
		Week 10	94.74 ± 2.8^ɣ^	125.8 ± 4.9^*c*^	101.1 ± 6.1^ɣ^	105.5 ± 4.6 ^ɣ^	103.3 ± 1.7^ɣ^
		Week 12	95.8 ± 1.8^ɣ^	127.6 ± 4.3^*c*^	96.6 ± 2.4^ɣ^	100.3 ± 2.2^ɣ^	93.3 ± 3.4^ɣ^
DBP (mmHg)	Induction period	Week 1	80.8 ± 2.2	70.4 ± 2.2	72.4 ± 3.9	77.9 ± 2.4	78.3 ± 3.7
		Week 3	76.6 ± 4.1	81.6 ± 1.3	90.8 ± 2.8^*b*^	75.5 ± 3.2	89.9 ± 6.5^*a*^
		Week 6	79.5 ± 1.9^β^	101.6 ± 3.0^*b*^	101.4 ± 2.2^*b*^	99.2 ± 4.3^*b*^	90.8 ± 3.7
	Treatment period	Week 8	78.1 ± 2.6^ɣ^	111.7 ± 5.7^*c*^	97.7 ± 7.8^*b*^	93.4 ± 6.9^α^	88.9 ± 4.8^*b*, ɣ^
		Week 10	79.4 ± 3.1^ɣ^	113.4 ± 6.1^*c*^	86.5 ± 7.4^*d*, ɣ^	88.9 ± 2.8^ɣ^	88.71 ± 2.3^ɣ^
		Week 12	81.9 ± 3.0^ɣ^	114.2 ± 5.2^*c*^	81.5 ± 4.9^α, ɣ^	83.5 ± 1.8 ^ɣ^	80.1 ± 4.5 ^ɣ^

Effect of administration of *A. officinarum (AO)* on systolic blood pressure, diastolic blood pressure, and mean blood pressure in obesogenic diet-fed hypertensive rats. The results are stated as means ± S.E.M (n = 6), where ^a=^ *p* < 0.05, ^b=^ *p* < 0.01, and ^c=^ *p* < 0.001 vs normotensive rats. ^α=^ *p* < 0.05, ^β=^ *p* < 0.01, and ^ɣ=^ *p* < 0.001 vs hypertensive rats.

**TABLE 5:** Effect of administration of *Alpinia officinarum on* heart rate (HR) beats per minute in chronic obesogenic diet-fed hypertensive rats.

Groups	Induction period			Treatment period		
	Wk1	Wk3	Wk6	Wk8	Wk10	Wk12
Normotensive	332.3 ± 3.9	337.8 ± 5.8	340.9 ± 9.6^ɣ^	346.2 ± 8.2^ɣ^	349.8 ± 4.4^ɣ^	355.2 ± 6.7^ɣ^
Hypertensive	327.4 ± 4.8	351.8 ± 7.7	405.4 ± 9.8^*c*^	454.0 ± 6.7^*c*^	460.5 ± 6.6^*c*^	466.2 ± 5.8^*c*^
Atorvastatin	331.4 ± 6.1	366.8 ± 8.0^*a*^	431.6 ± 5.8^*c*^	420.7 ± 5.7^*c,*^ ^β^	381.8 ± 7.3 ^*b*, ɣ^	364.2 ± 7.3^ɣ^
AO (250 mg/kg)	326.5 ± 5.7	376.1 ± 4.5^*c*^	450.4 ± 5.8^*c,*^ ^ɣ^	410.6 ± 9.8^*c*,ɣ^	380.5 ± 5.9^*a*,ɣ^	378.8 ± 3.6^ɣ^
AO (500 mg/kg)	330.5 ± 9.6	390.8 ± 7.7^*c*,ɣ^	442.6 ± 6.4^*c*, β^	400.7 ± 9.5^*c*, ɣ^	386.8 ± 4.5 ^*b*, ɣ^	370.4 ± 7.7^ɣ^

Effect of administration of *A. officinarum (AO) on* heart rate (HR) beats per minute in chronic obesogenic diet-fed hypertensive rats. The results are stated as means ± S.E.M (*n* = 6), where ^a=^ *p* < 0.05, ^b=^ *p* < 0.01, and ^c=^ *p* < 0.001 vs normotensive rats. ^α=^ *p* < 0.05, ^β=^ *p* < 0.01, and ^ɣ=^ *p* < 0.001 vs hypertensive rats.

### Effect of *A. officinarum* on Serum Biochemical Parameters

The effects of AO extract on the biochemical lipid profile of treated rats are presented in Table 6. The hypertensive control group displayed a significant increase in serum TC, LDL-C, and TG except for HDL-C, while all treated groups (AO 250 and 500 mg/kg) showed an insignificant variation when compared to normotensive rats. In treated obese hypertensive rats, the administration of AO (both doses) exhibited a significant reduction (*p* < 0.001) in the serum lipid components (TC, LDL-C, TG) when compared to the hypertensive control data, while HDL-C was found slight raised; similar effects were observed on the part of atorvastatin (10 mg/kg) as seen in Table 6.

**TABLE 6:** Effect of administration of *Alpinia officinarum on serum biochemical parameters* in chronic obesogenic diet-fed hypertensive rats.

Groups	ALT (IU/L)	AST (IU/L)	ALP (IU/L)<	Bilirubin (mg/dl)	Albumin (g/dl)	Total protein (g/dl)	Urea (mg/dl)	Creatinine (mg/dl)	Total cholesterol (TC) (mg/dl)	HDL cholesterol (mg/dl)	LDL cholesterol (LDL_C) (mg/dl)	Triglyceri-des (TG) (mg/dl)
Normotensive	71.1 ± 1.5^ɣ^	138.1 ± 6.9^ɣ^	156.0 ± 5.8^ɣ^	0.8 ± 0.1^ɣ^	2.2 ± 0.1^ɣ^	4.7 ± 0.2^ɣ^	31.0 ± 4.7^β^	0.8 ± 0.1^ɣ^	90.2 ± 2.5^ɣ^	42.9 ± 2.8	25.8 ± 1.3^ɣ^	105.2 ± 4.3^ɣ^
Hypertension	181.4 ± 6.1^*c*^	184.3 ± 4.3^*c*^	199.3 ± 5.7^*c*^	1.5 ± 0.1^*c*^	1.8 ± 0.1^*c*^	3.1 ± 0.1^*c*^	58.5 ± 5.7^*b*^	2.0 ± 0.1^*c*^	154.2 ± 3.1^*c*^	34.4 ± 3.0	59.1 ± 2.2^*c*^	256.4 ± 8.9 ^*c*^
Atorvastatin	73.3 ± 5.8^ɣ^	142.3 ± 4.8^ɣ^	164.6 ± 7.0^ɣ^	1.1 ± 0.1^β^	2.2 ± 0.1^β^	4.6 ± 0.1^ɣ^	35.2 ± 5.7^α^	1.0 ± 0.1^ɣ^	92.3 ± 3.6^ɣ^	41.0 ± 2.5	29.7 ± 1.7^ɣ^	112.5 ± 2.5^ɣ^
AO (250 mg/kg)	79.2 ± 4.0^ɣ^	159.3 ± 5.8^α^	171.2 ± 6.1^β^	1.0 ± 0.1^ɣ^	2.2 ± 0.1^β^	4.6 ± 0.0^ɣ^	41.1 ± 5.8	1.1 ± 0.0 ^ɣ^	126.2 ± 3.6^*c*, ɣ^	35.9 ± 3.7	42.7 ± 2.6^*c*, ɣ^	138.5 ± 4.3^*c*, ɣ^
AO (500 mg/kg)	74.4 ± 5.8^ɣ^	145.2 ± 5.8^ɣ^	163.5 ± 6.8^ɣ^	0.9 ± 0.0^ɣ^	2.2 ± 0.1^β^	4.6 ± 0.0^ɣ^	35.3 ± 5.8^α^	0.9 ± 0.1^ɣ^	89.2 ± 2.0^ɣ^	41.4 ± 3.7	31.3 ± 2.8^ɣ^	116.2 ± 3.4^ɣ^

Effect of administration of *Alpinia officinarum on serum biochemical parameters* in chronic obesogenic diet-fed hypertensive rats. The results are stated as means ± S.E.M (n = 6), where ^a=^ *p* < 0.05, ^b=^ *p* < 0.01, and ^c=^ *p* < 0.001 vs normotensive rats. ^α=^ *p* < 0.05, ^β=^ *p* < 0.01, and ^ɣ=^ *p* < 0.001 vs hypertensive rats.

The treatment groups (AO, 250 and 500 mg/kg) also revealed a marked reduction of ALT, AST, ALP, and bilirubin compared to that of hypertensive obese animals. An increase in serum albumin and total protein was observed at tested doses of the plant extract (AO, 250 and 500 mg/kg) and atorvastatin in comparison with hypertensive obese rats. AO (250 and 500 mg/kg) administration to rats exerted a noticeable (*p* < 0.001) decrease in serum urea and creatinine levels compared to hypertensive rats (Table 6).

**FIGURE 2:**
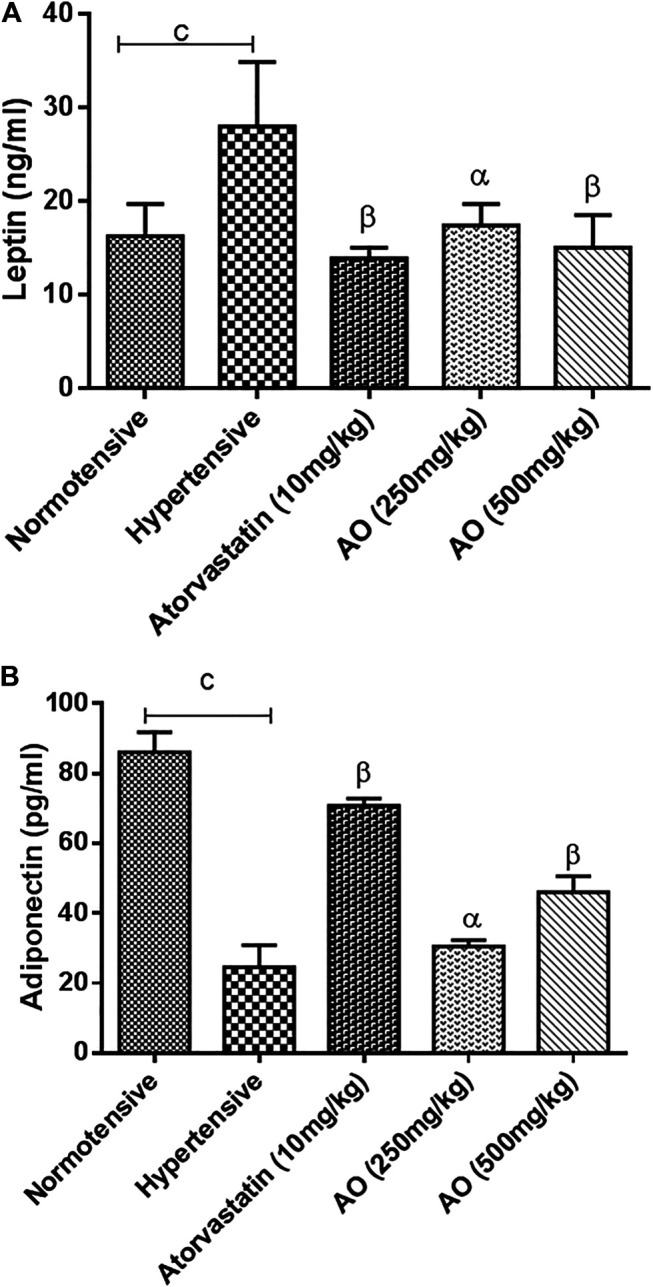
Effect of administration of *A. officinarum (AO* *)* on obesity biomarkers, **(A)** leptin and **(B)** adiponectin in chronic obesogenic diet-fed hypertensive rats. Normotensive group = Standard diet, hypertensive group = High fat, sucrose, and salt (HFSS) diet, atorvastatin group = HFSS + atorvastatin (10 mg/kg/day in normal saline, per oral), AO (250 mg/kg, per oral) group = HFSS + AO (500 mg/kg, per oral) and AO (500mg/kg) group = HFSS + AO (500mg/kg). The results are stated as means ± S.E.M (n = 6). ^b=^ *p* < 0.01 and ^c=^ *p* < 0.001 vs normotensive rats. ^α=^ *p* < 0.05, ^β=^ *p* < 0.01, and ^ɣ=^ *p* < 0.001 vs hypertensive rats.

The hypertensive group showed a high (*p* < 0.01) amount of serum leptin compared to normotensive rats. In the treated groups, AO extract (250 and 500 mg/kg) and atorvastatin (10 mg/kg) brought down (*p* < 0.01) the serum leptin levels towards normal after 6 weeks of treatment vs. hypertensive rats (Figure 2A). However, a significant decrease (*p* < 0.001) in adiponectin levels was observed in the hypertensive rats vs normotensive rats. AO extract (250 and 500 mg/kg) or atorvastatin (10 mg/kg) treatment elevated (*p* < 0.001) the reduced levels of adiponectin compared to hypertensive rats (Figure 2B).

#### Effect of *A. officinarum* on Oxidative Stress

**FIGURE 3:**
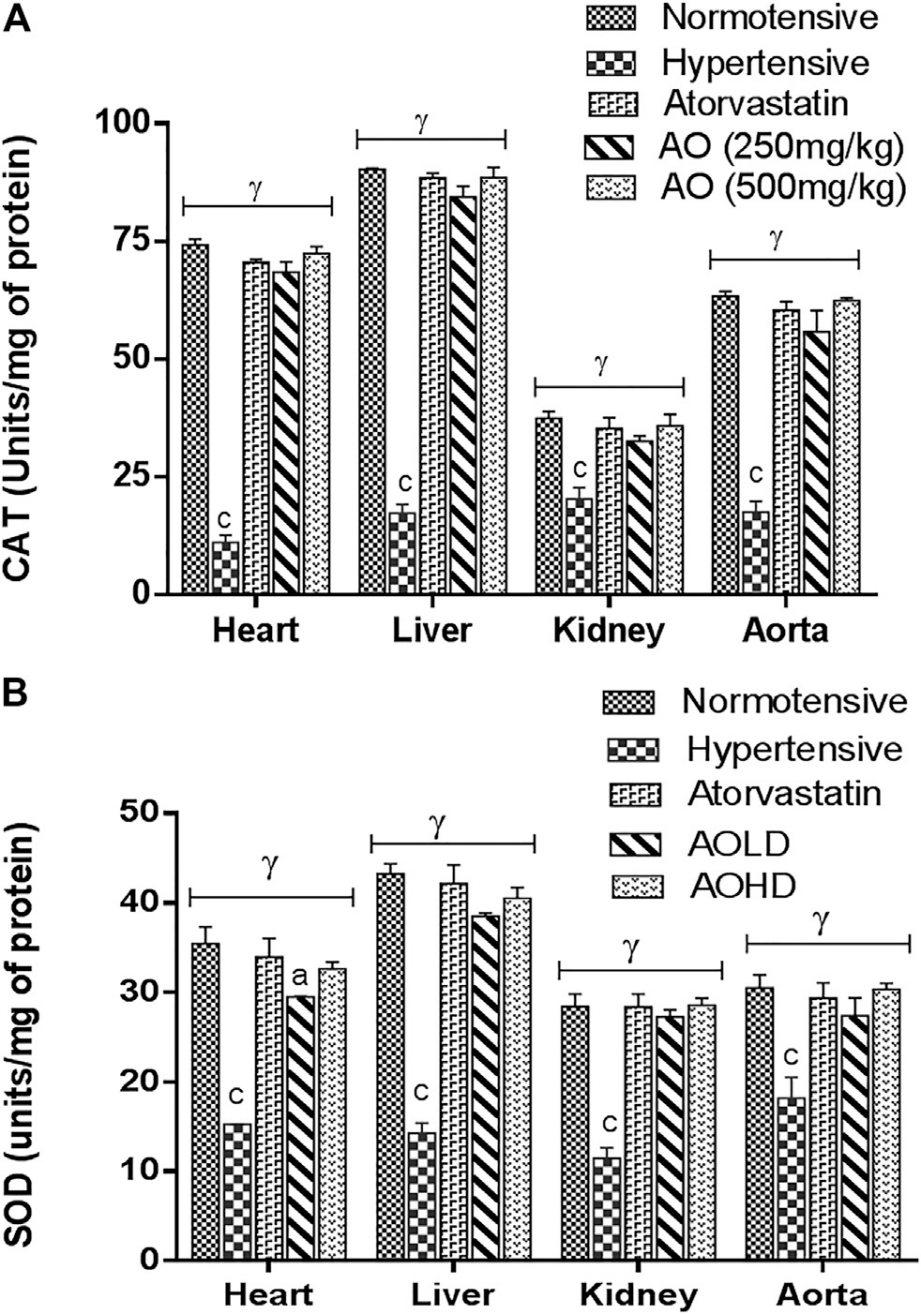
Effect of administration of *A. officinarum (AO)* on **(A)** catalase (CAT) and **(B)** superoxide dismutase (SOD) enzyme in various organs of chronic obesogenic diet-fed hypertensive rats. The results are stated as means ± S.E.M (*n* = 6), where ^a=^ *p* < 0.05, ^b=^ *p* < 0.01, and ^c=^ *p* < 0.001 vs normotensive rats. ^α=^ *p* < 0.05, ^β=^ *p* < 0.01, and ^ɣ=^ *p* < 0.001 vs hypertensive rats.

Figure 3 represents the oxidative stress marker in tissues of the heart, liver, kidney, and aorta. A marked decrease (*p* < 0.001) of production of antioxidant enzyme (CAT and SOD) status was observed in the tissues of hypertensive vs normotensive rats. The groups treated with extracts of AO and atorvastatin raised (*p* < 0.001) the antioxidant enzyme (CAT and SOD) activity compared to hypertensive rats (Figure 3).

#### Effect on Histopathology of Organs

**FIGURE 4:**
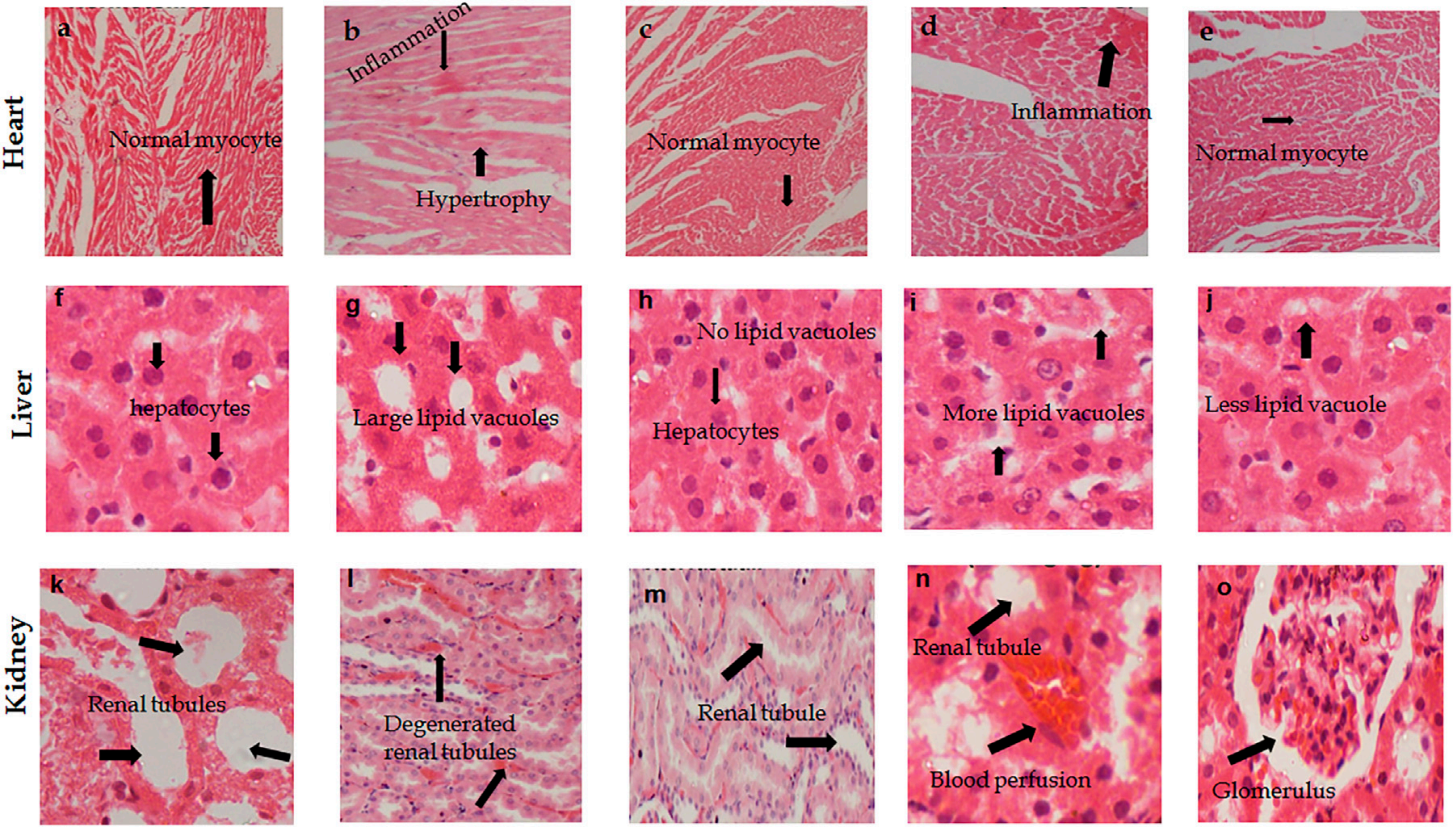
Photomicrographs H & E stained tissue sections of heart, kidney, and liver showing the effect of (*A. officinarum*) AO treatment in chronic obesogenic diet-fed hypertensive rats. **(A, F, K)** Normotensive rats, **(B, G, and I)** hypertensive rats = high fat, sucrose, and salt (HFSS) diet, **(C, H, and M)** HFSS + atorvastatin (10 mg/kg, per oral)-treated rats, **(D, I, and N)** HFSS + AO (250 mg/kg, per oral)-treated rats **(E, J, and O)** HFSS + AO (500 mg/kg, per oral) treated rats. 400 X, scale bar displays 50 μm.

The histopathological analysis of the liver of all groups of rats showed normal anatomical features including the normal form of liver lobules and the central vein with a smaller number of food vacuoles in treated groups compared to hypertensive rats. Rat hearts in the treated groups showed normal myocytes and minimal to no inflammation compared to hypertensive rats. Whereas, the renal tubules of all treated rats showed minimal to no inflammation (Figure 4).

### Effect of *A. officinarum* on Diuresis

The extract of AO depicted dose-dependent diuretic activity at both tested doses compared to the hypertensive group at the 5th and 24th h. The aggregate of urinary output at the 5th and 24th h was noted for the normotensive, hypertensive, and treatment groups (furosemide, at 10 mg/kg and hydroethanolic *A. officinarum* ethanolic extracts at 250 and 500 mg/kg). Treatment with *A. officinarum* enhanced (*p* < 0.05) diuresis similar to the effect of furosemide. The diuretic activity of *A. officinarum* extract was mild because it ranged from 0.72 to 1 (Table 7).

**TABLE 7:** Effect of administration of *Alpinia officinarum* on urine volume of chronic obesogenic diet-fed hypertensive rats at 5 and 24 h intervals on the last day of the model.

Groups	At 5 h after administration of drug			At 24 h after administration of drug		
	Urine volume (ml)	Diuretic action	Diuretic activity	Urine volume (ml)<	Diuretic action	Diuretic activity
Normotensive (N/S 2 ml/100g)	1.4 ± 0.0 ^ɣ^	1	—	5.5 ± 0.1^α^	1	—
Hypertensive (N/S 2 ml/100g)	2.3 ± 0.1^*c*^	1.7	0.6	4.5 ± 0.1^a^	0.8	0.4
Furosemide hypertensive (10 mg/kg)	4.1 ± 0.0 ^*c*,ɣ^	3.0	1.0	10.4 ± 0.2^*c*, ɣ^	1.9	1
AO (250 mg/kg)	3.5 ± 0.1^*c*,ɣ^	2.7	0.8	6.1 ± 0.2^*b*, β^	1.1	0.6
AO (500 mg/kg)	3.9 ± 0.2^*c*,ɣ^	2.9	0.9	8.3 ± 0.1^*c*, ɣ^	1.5	0.8

Effect of administration of *Alpinia officinarum* on urine volume of chronic obesogenic diet-fed hypertensive rats at 5 and 24 h intervals on the last day of the model. Diuretic action = urine output of the treated group/urine output of the normotensive group. Diuretic activity = urine output of the treated group/urine output of the furosemide group. The results are stated as means ± S.E.M (n = 6), ^a=^ *p* < 0.05, ^b=^ *p* < 0.01, and ^c=^ *p* < 0.001 vs normotensive rats. ^α=^ *p* < 0.05, ^β=^ *p* < 0.01, and ^ɣ=^ *p* < 0.001 vs hypertensive rats.

#### Effect of *A. officinarum* on pH and Conductivity of Urine

The pH and conductivity of urine samples of normotensive (pH: 6.5 ± 0.1, conductivity: 14.0 ± 1.2, *n* = 6) vs hypertensive (6.2 ± 1.1, 9.3 + 1.1, *n* = 6) rats showed a significant (*p* < 0.05) difference in only conductivity values. Furosemide (10 mg/kg, p. o) and AO (250 and 500 mg/kg) treatment to rats caused a significant rise in pH and conductivity compared to hypertensive rats with respective values of 7.4 ± 0.1, 32.0 + 0.7 vs 6.2 ± 1.1, 9.3 + 1.1, (*p* < 0.001), 7.0 ± 0.1, 17.0 + 2.7 vs 6.2 ± 1.1, 9.3 + 1.1, (*p* < 0.01), and 7.3 ± 0.1, 27.6 + 2.1 vs 6.2 ± 1.1, 9.3 + 1.1, (*p* < 0.001), respectively, thus shifting the pH of urine to slightly alkaline.

#### Effect of *A. officinarum* on Urinary Electrolyte Content

The urinary electrolyte contents of the hydroethanolic *A. officinarum* extract- and furosemide-treated groups were higher (*p* < 0.05) than hypertensive rats. The AO extract at low and high doses revealed an increment in urinary Na^+^, K^+^, and Cl^−^ ion excretion, similar to the effect of furosemide-treated rats (Table 8).

**TABLE 8:** Effect of administration of *Alpinia officinarum* on urinary electrolytes of chronic obesogenic diet-fed hypertensive rats at 24 h on the last day of the model.

Groups	Na^+^ (mg/L)	K^+^ (mg/L)	Cl^−^ (mg/L)	Na^+^ index	K^+^ index	Cl index
Normotensive (N/S 2 ml/100g)	116.2 ± 5.5^ns^	62.1 ± 1.5^ns^	64.0 ± 3.1^ns^	1	1	1
Hypertensive (N/S 2 ml/100g)	108.0 ± 1.9^ns^	71.0 ± 1.3^ns^	62.3 ± 1.2^ns^	0.9	1.1	0.9
Furosemide hypertensive (10 mg/kg)	194.0 ± 1.5^*c*, ɣ^	94.3 ± 5.1^*c*, ɣ^	90.7 ± 2.0 ^ɣ^	1.7	1.5	1.4
AO (250 mg/kg)	145.0 ± 4.83^*c*, ɣ^	80.3 ± 1.3^*b*,ns^	64.3 ± 2.9^ns^	1.2	1.3	1.0
AO (500 mg/kg)	174.0 ± 2.8^*c*, ɣ^	85.0 ± 1.8^*c*, β^	71.0 ± 3.6^ns^	1.5	1.4	1

Effect of administration of *Alpinia officinarum* on urinary electrolytes of chronic obesogenic diet-fed hypertensive rats at 24 h on the last day of the model. Na^+^ = sodium; K^+^ = potassium; Cl-= chloride. The results are stated as means ± S.E.M (*n* = 6), ^ns^ = non-significant, ^a=^ *p* < 0.05, ^b=^ *p* < 0.01, and ^c=^ *p* < 0.001 vs normotensive rats.^α=^ *p* < 0.05, ^β=^ *p* < 0.01, and ^ɣ=^ *p* < 0.001 vs hypertensive rats.

#### Natriuretic (Na^+^/K^+^), Saluretic (Na^+^+Cl^−^), and Carbonic Anhydrase Inhibition (CAI) Effects of *A. officinarum*

The hydroethanolic extract of *A. officinarum* at 250 and 500 mg/kg depicted high (*p* < 0.05) saluretic activity and natriuretic response and strong CAI activity compared to hypertensive rats, similar to the effect of furosemide-treated animals (Table 9).

**TABLE 9:** Effect of administration of *Alpinia officinarum* on saluretic effect, natriuretic effect, and carbonic anhydrase inhibition of chronic obesogenic diet-fed hypertensive rats at 24 h on the last day of the model.

Groups	Saluretic effect (Na^+^ + Cl^−^)	Natriuretic effect (Na^+^/K^+^)	CAI [Cl/(Na^+^+K^+^]	Saluretic index	Natriuretic index	CA index
Normotensive (N/S 2 ml/100g)	179.7 ± 5.3^α^	1.8 ± 0.0^ns^	0.4 ± 0.0^ns^	1	1	1
Hypertensive (N/S 2 ml/100g)	170.3 ± 2.4^*a*^	1.5 ± 0.1^ns^	0.4 ± 0.0^ns^	0.9	0.8	0.97
Furosemide hypertensive (10 mg/kg)	284.7 ± 1.7^*c*, ɣ^	2.1 ± 0.1^ns^	0.3 ± 0.0^ns^	1.5	1.1	0.8
AO (250 mg/kg)	209.3 ± 6.7^*c*^ ^ɣ^	1.8 ± 0.1^ns^	0.3 ± 0.0^ns^	1.2	1.0	0.8
AO (500 mg/kg)	245.0 ± 3.6^*c*, ɣ^	2.1 ± 0.1^ns^	0.3 ± 0.0^ns^	1.4	1.1	0.7

Effect of administration of *Alpinia officinarum* on saluretic effect, natriuretic effect, and carbonic anhydrase inhibition of chronic obesogenic diet-fed hypertensive rats at 24 h on the last day of the model. Na^+^ = sodium; K^+^ = potassium; Cl-= chloride. The results are stated as means ± S.E.M (*n* = 6), ^ns^ = non-significant, ^a=^ *p* < 0.05, ^b=^ *p* < 0.01, and ^c=^ *p* < 0.001 vs normotensive rats. ^ns^ = non-significant, ^α=^ *p* < 0.05, ^β=^ *p* < 0.01, and ^ɣ=^ *p* < 0.001 vs hypertensive rats.

### Gene Expression Alterations

**FIGURE 5:**
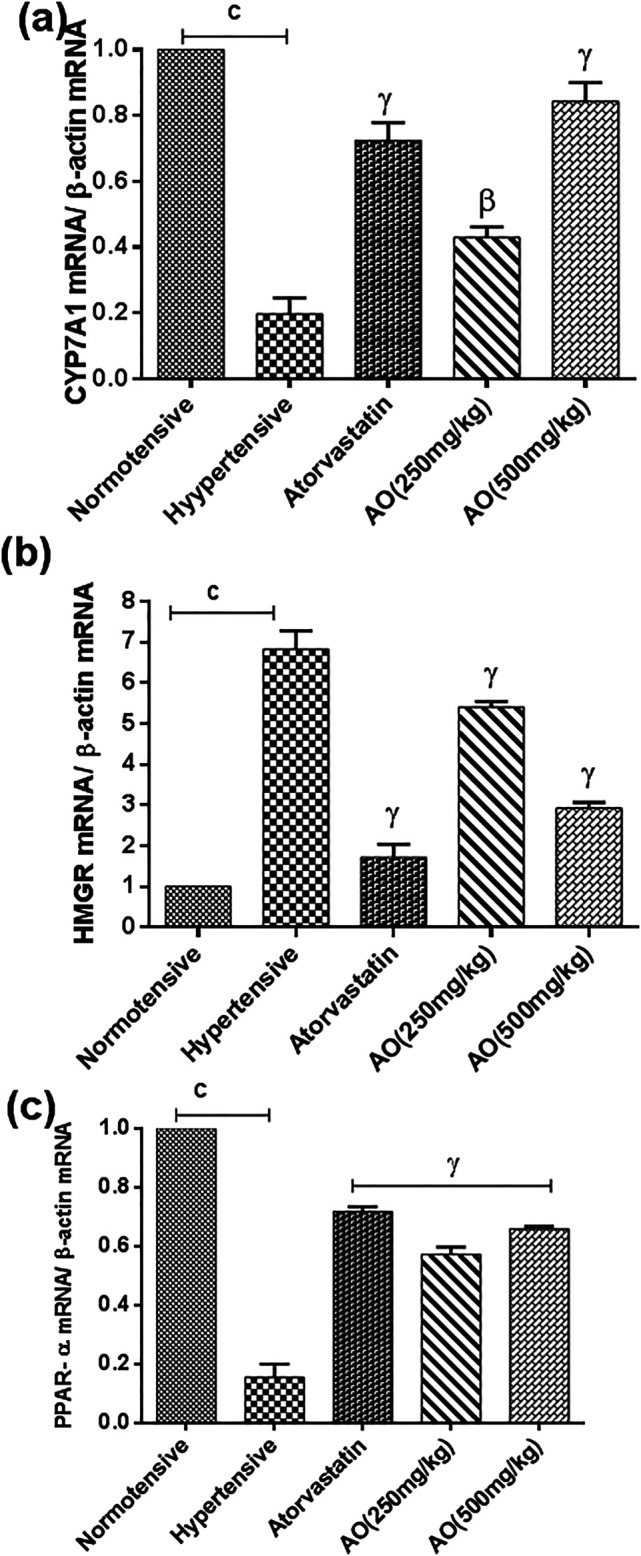
Effect of administration of *A. officinarum (AO)* on mRNA expression of **(A)** 7α-hydroxylase (CYP7A1), **(B)** 3-hydroxy-3methyl-glutaryl-coenzyme A reductase (HMGR), and **(C)** peroxisome receiver activator alpha (PPAR-α) performed by qRT-PCR in the liver of chronic obesogenic diet-fed hypertensive rats. The results are stated as means ± S.E.M (*n* = 6), where ^a=^ *p* < 0.05, ^b=^ *p* < 0.01, and ^c=^ *p* < 0.001 vs normotensive rats. ^α=^ *p* < 0.05, ^β=^ *p* < 0.01, and ^ɣ=^ *p* < 0.001 vs hypertensive rats.

The triplicate samples from each group produced reproducible results. Relative to the normotensive group, the hypertensive group significantly decreased the expression of genes encoding peroxisome proliferator-activated receptor (PPAR)-alpha (0.15 ± 0.03 folds vs 1.00 ± 0.00 normotensive) and cholesterol 7 alpha-hydroxylase (CYP7A1) (0.20 ± 0.05 folds vs 1.00 ± 0.00 normotensive) while increased the expression of gene encoding 3-hydroxy-3-methylglutaryl-CoA reductase (HMGR) (6.83 ± 0.45 folds vs 1.00 ± 0.00 normotensive) (*p* < 0.001, Figure 5). However, administration of atorvastatin and AO (HFSS + atorvastatin and HFSS + AO 250 mg/kg, HFSS + AO 500 mg/kg) significantly increased the gene expression of *PPAR-alpha* (0.72 ± 0.02 folds, 0.57 ± 0.02 folds, and 0.66 ± 0.01 folds, respectively) and *CYP7A1* (0.72 ± 0.05 folds, 0.43 ± 0.03 folds, and 0.84 ± 0.06 folds, respectively) while decreased the mRNA expression of *HMGR* (1.73 ± 0.31 folds, 5.41 ± 0.14 folds, and 2.93 ± 0.14 folds, respectively) when compared to obesity-induced hypertensive rats (HFSS group) (*p* < 0.001, Figure 5).

## Discussion

Hypertension influences 40% of people in the world and is considered a major health issue (Feng et al., 2013). *A. officinarum* is used for the improvement of the cardiovascular system and the genus is famous for a significant reduction in blood pressure (Victório, 2011; Manosroi et al., 2013). In this study, we evaluated the antihypertensive, antihyperlipidemic, biomarkers of obesity (leptin and adiponectin), and diuretic effects of the hydroethanolic extract of *A. officinarum* in obesogenic feed-induced hypertensive rats. After the induction period of the first 6 weeks, the results revealed a significant increase in blood pressure and weight gain compared to normotensive rats.

The high caloric model was designed to imitate third-world diets that usually depict excessive carbohydrate, fat, and salt consumption. The hyper-caloric diet used in the project was of an adequate intensity and period to stimulate obesity in rats. Ingestion of high caloric feed has been linked with various feed-induced issues such as obesity, oxidative stress, hyperlipidemia, and hypertension (Huang et al., 2011; Ragab et al., 2015). Anthropometric factors comprise important markers for the diagnosis of obesity. Among these factors, body weight, Lee index, and weight of various organs (heart, left ventricle, liver, and kidney) were assessed. In the last week of the induction period (first 6 weeks of the model), increment in the mass of the body was seen in all groups compared to normotensive rats. It has also been documented in many articles that a high caloric diet causes a rapid increase in weight gain. While at the end of treatment (at the 12th week), the data revealed that, oral administration of AO (250 and 500 mg/kg) significantly reduced weight gain, the weight of various organs, and the Lee index in the experimental rats compared to the hypertensive group. This reduction was comparable to atorvastatin, as the statin family is known to induce weight loss in obese subjects via inhibition of gastric and pancreatic lipase, and reduction in the absorption of dietary fat (Xia et al., 2010; Garg and Singh, 2015). The hypertensive group showed the highest weight gain throughout the model which is also supported by an earlier study (Xia et al., 2010; Yao et al., 2015).

The significant reduction in Lee index was seen in atorvastatin- and AO- (250 and 500 mg/kg) treated rats and it could be related to the loss in body weight gain of AO-treated rats, which is also similar to earlier reports in the literature (Xia et al., 2010; Cavagni et al., 2013). The presence of galangin, kaempferol, kaempferide, and quercetin as active constituents have earlier been documented (Matsuda et al., 2009; Zhang et al., 2015) where the flavonoids are known to cause a slimming effect (F. Chen et al., 2013).

There is increasing evidence supporting the fact that diet induces obesity which in turn causes hypertension. Resultant hypercholesterolemia, vascular endothelial dysfunction, and visceral fats deposition are said to be the indices of inflammation, cardiac hypertrophy, and cardiac fibrosis (Dobrian et al., 2000; V.; Kotsis et al., 2010; Panchal and Brown, 2010). However, this is the first study reporting the blood pressure lowering efficacy of *A. officinarum* in obesity-induced hypertensive rats. In the termination of the induction period, SBP, MBP, DBP, and HR were significantly raised in the rats of all groups except in normotensive rats, while at the 12th week, the treatment groups showed comparable values to the normotensive rats except the animals of the hypertensive group. The reduction in SBP, MBP, DBP, and HR with hydroethanolic extract of AO indicates it may be a potential candidate in the management of obesity-induced cardiac complications. These findings also provide a rationale for the medicinal use of *A. officinarum* as a cardiotonic or hypotensive agent (Basri et al., 2017). The blood pressure lowering effects are also in agreement with the prior findings related to vasorelaxant effects of *Alpinia zerumbet* (Lahlou et al., 2003), which might play a pivotal role in the overall effectiveness of this plant in hypertension.

HFSS feed administration-developed dyslipidemia showed a high amount of TG, TC, and LDL-C, while there was a decreased level of HDL-C in rats compared to normotensive animals. These results may be due to the increased amount of fat (obtained from beef tallow) which induced hypercholesterolemia (Cho et al., 2010; Kelany et al., 2017). An increase in total cholesterol or LDL-C is a predisposing condition for the development of cardiovascular complications including hypertension (Yang et al., 2006). However, the intake of dietary polyphenols efficiently decreases the amount of triglyceride-rich lipoprotein and is linked to oxidative stress in fasting and postprandial conditions (Annuzzi et al., 2014).

In this study, the treatment groups showed appreciable catalase and superoxide dismutase enzymatic activities compared to hypertensive rats. Catalase is an enzyme with ferric heme and contributes to lower the amount of hydrogen peroxide by converting it into water and molecular oxygen. So in the case of a decrease in the amount of catalase, hydrogen peroxide can accumulate which in turn forms highly reactive hydroxyl free radicals. In this study, the high amount of the enzyme in the treated groups can be attributed to antioxidants in the plant extract. These results are also similar to another study (Zhan et al., 2004). Nitric oxide (NO) plays an important role in the stabilization of blood pressure. Free radicals like superoxide quickly react with NO and in turn produce highly reactive and cytotoxic compounds like peroxynitrite and peroxynitrous acid. Furthermore, peroxynitrite changes many compounds including lipids, deoxyribonucleic acid, and proteins. It also reacts with amino acids such as tyrosine and cysteine to form nitrotyrosine and nitrocystein, causing the deactivation of superoxide dismutase (Alvarez et al., 2004). The presence of important dietary polyphenol and flavonoids such as gallic acid, quercetin, and kaempferol were confirmed as plant constituents by performing HPLC. The presence of such valuable constituents also contributes to the assessed beneficial effects of *A. officinarum* in obesity-related cardiac issues*.*

Leptin and adiponectin are biologically active secretions from an important endocrine gland in adipose tissue. Leptin plays a cardinal role in balancing feed intake and weight of the body while its level is majorly affected by dietary fat (Kim et al., 2007). A high amount of leptin is released from large sized adipose tissue of obese rats that act as an indicator to the central nervous system revealing the volume of power reserves (Nagao et al., 2003). The recent finding suggested that endothelial dysfunction is linked with hypoadiponectinemia and causes diet-induced hypertension (Ouchi et al., 2003). The hydroethanolic extract of *A. officinarum* ameliorated the lipid contents by decreasing serum TC, TG, LDL-C, and leptin contents, while serum adiponectin and HDL-C concentrations were found to increase when compared with the hypertensive rats. These results suggested that *A. officinarum* possesses the potential to protect against hypertension in obese rats.

Followed by an injury to the hepatic cells, the permeability of the plasma membrane is enhanced which in turn causes the release of enzymes from hepatic cells. The level of aminotransferase, ALP, and bilirubin are raised in the serum in case of liver injury. Biliary tract obstruction is indicated by the high amount of ALP and bilirubin in serum while the increased amounts of ALT and aspartate aminotransferase are characteristic indicators of liver injury (Saleem et al., 2014). The hydroethanolic extract of *A. officinarum* improved the liver and kidney indices of toxicity such as ALT, AST, ALP, bilirubin, total protein, albumin, creatinine, and urea levels.

The histopathological data revealed that the hearts of the hypotensive group of rats showed an abnormal texture of myocytes and blood perfusion. While AO extract- and atorvastatin-treated rats showed the normal appearance of myocytes with no blood perfusion. Histograms of the liver sections of hypertensive rats indicated degenerated hepatocytes and many vacuoles, while AO extract treatment showed normal hepatocytes and less vacuolation compared to hypertensive rats. In the case of kidney tissue sections, the hypertensive group showed degenerated renal tubules and inflammation, while AO treatment showed a revived (similar to normal) form of renal tubules.

The diuretic response of *A. officinarum* was assessed and findings showed that *A. officinarum* (250 and 500 mg/kg) enhanced urine volume over a time period of 5 and 24 h. The diuretic activity of the AO extract was comparable to the furosemide group. The data revealed that the AO extract exerted time- and dose-dependent diuretic effects. It has earlier been known that the increase in the volume of urine of rodents may have resulted from the increased amount of K^+^ ions in the plant (Nilvises et al., 1989). The pH of animals administered with AO extract was alkaline when compared with the hypertensive group. However, the pH of the treatment groups (AO extract) was comparable to the furosemide-treated group. The AO extract (250 and 500 mg/kg) enhanced the excretion of ions (Na^+^, K^+^, and Cl^−^) in urine than in the hypertensive group. This activity might be due to the combined effect of the (HCO3^_^/Cl^−^) (HCO3+/H+), and the (Na+/H+) antiporter, causing diuresis. This is a cardinal feature of loop diuretics. Furosemide shows its effect by decreasing ion reabsorption in the thick loop of Henle (Al-Saikhan and Ansari, 2016). While the hypertensive group showed the least urinary output and it is supported by a study in which obese rats showed less urinary output compared to normal rats (Ansari, 2015). A high amount of body fat stimulates the synthesis of angiotensinogen and angiotensinogen is then converted into angiotensin II in the presence of renin. This high amount of angiotensin II is responsible for less water excretion and more tubular reabsorption of electrolytes (sodium and chloride ions) (Kotsis et al., 2010). After oral administration of AO extract, there was increased excretion of water from obese hypertensive rats. This increase could be justified by the active constituent of the AO extract (almost 90 ethno-medicinal ingredients have been obtained from the AO extract). Phenolics are majorly identified, particularly diarylheptanoids separated from the rhizome of *A. officinarum,* and are supposed to be the most effective biologically active constituents (Abubakar et al., 2018). There is a need to check the effect of isolated components of AO on diuresis.

The ratio between sodium and potassium ions is the measure of natriuretic activity. If the ratio is greater than 2.0, it indicates a high natriuretic effect and if the ratio becomes 10.0, it represents a potassium-sparing effect (Vogel and Vogel, 2013). In this study, Na^+^/K^+^ values of the AO extract were calculated. It indicated a natriuretic effect of more than two that supported the strong natriuretic effect of the AO extract at a high dose. A value of more than two indicated that there was more sodium excretion than potassium in the AO extract-treated group (500 mg/kg), which confers a diuretic effect with a satisfactory safety profile. Mostly synthetic diuretics cause the adverse effect of severe hypokalemia.

The Cl^−^/(Na^+^+K^+^) ratio revealed the level of carbonic anhydrase inhibition (CAI) response; CAI effect is considered negligible at ratios between 1.0 and 0.8, while a ratio less than 0.8 indicates CAI activity in the test material (Vogel and Vogel, 2013). The amount of CAI was calculated for low and high doses of AO extract and they showed a ratio less than 0.8 at 0.28 and 0.27, respectively. It showed the strongest CA inhibitory effect of AO. There is the possibility that the most effective pathway for diuresis of the AO extract could be due to CA inhibition.

The protein, peroxisome receiver activator alpha (PPAR-α), has an important role in energy metabolism (hence its role as a therapeutic target for dyslipidemia) and is currently known as a modulator of inflammation. However, the exact role of PPAR-α in cholesterol metabolism is yet unclear, although some studies have proposed its role in reverse cholesterol transport and regulation of HDL metabolism. According to the studies, fibrate (agonist of PPAR) causes an increase in the level of HDL, decreases triglycerides, and minimally reduces the amount of LDL (de Miranda et al., 2017). The level of *PPAR*-*α* mRNA was lower in obese hypertensive rats when compared to normotensive rats. While treatment with AO and atorvastatin showed a significant increase in the level of *PPAR*-*α* mRNA in liver tissue which is in accordance with a previous study (Stec et al., 2019). For endogenous cholesterol biosynthesis, HMGR is considered as a rate-limiting enzyme and it catalyzes conversion of the HMG-CoA into mevalonate. HMG-CoA reductase inhibitors (a class of drugs) and statins are used clinically as the first line of treatment for the reduction in the level of mevalonate (metabolite) in serum (J. Chen et al., 2012). In our project, an increase was observed in the liver *HMGR* mRNA level in the HFSS diet-fed group of rats, supporting the fact that obesity is concomitant to increasing total cholesterol balance and increasing synthesis of cholesterol, majorly in the liver (Kalaivani et al., 2019). The expression level of *HMGR* in the HFSSD group was considerably downregulated by AO or atorvastatin administration and hence synthesis of cholesterol was highly decreased by reducing total cholesterol and LDL-C in plasma. Many drugs that have the potential to lower the total cholesterol level in the plasma block the functional site of the HMGR enzyme (Istvan, 2002). Compounds or protein in AO may inhibit the HMGR enzyme for the synthesis of cholesterol by binding at its active site and another study showed that medicinally active plants, containing flavonoids, have also been seen to decrease the activity of HMGR (Xie et al., 2007). The result suggested that AO has a similar pathway to that of statins. 7α-hydroxylase (Cyp7a1) plays a key role in the synthesis of bile acids (chenodeoxycholic acid and cholic acid). An enhanced level of mRNA *Cyp7a1* is responsible for the conversion of cholesterol to bile acids and hence, decreases hepatic free cholesterol and oxidative stress. Some studies reported that Cyp7a1 causes a very high bile acid pool but lacks triglycerides so protects against diet-induced obesity (Ferrell et al., 2019).

Many studies showed that the phytoconstituents of *A. officinarum,* phenolics (diarylheptanoids), flavonoids (3- methylethergalangin), coumaryl alcohol, and phenylpropanoids, are accountable for its multiple therapeutic activities (Abubakar et al., 2018). Flavonoids and phenolic compounds have the potential of many biological activities due to their antioxidant and free radical scavenging properties (Selamoglu, 2017). Furthermore, it is unclear which constituent is responsible for the blood pressure lowering and diuretic effect of *A. officinarum*. Hence, further investigations are needed to determine the biologically active constituent responsible for the hypotensive and diuretic activities of *A. officinarum*.

## Conclusion

This study shows that *A. officinarum* possesses antihypertensive and diuretic effects possibly mediated through attenuation of obesity markers (weight gain, leptin, and adiponectin), lipid parameters and appreciable diuretic, catalase, and superoxide dismutase activities. These data provide evidence to the conventional use of its decoction as an antihypertensive and diuretic agent.

## Data Availability

The original contributions presented in the study are included in the article/Supplementary Material, further inquiries can be directed to the corresponding author.

## Abbreviations

ALP, alkaline phosphatase; AO, *A. officinarum*; AST, aspartate aminotransferase; CA, carbonic anhydrase; CAI, carbonic anhydrase inhibition; CAT, catalase enzyme; DBP, diastolic blood pressure; ELISA kit, enzyme linked immunosorbent assay kit; HDL-C, high density lipoprotein cholesterol; HFSS, high fat-sugar-salt; HPLC, high-performance liquid chromatographic; HR, heart rate; LD50%, lethal dose in 50%; LDL-C, low density lipoprotein cholesterol; MBP, mean blood pressure; NIBP, noninvasive blood pressure measurement; SBP, systolic blood pressure; SD, standard diet; SOD, superoxide dismutase; TC, total cholesterol; TG, total glycerides.

## References

[B1] Abubakar I. B. Malami I. Yahaya Y. Sule S. M. (2018). A Review on the Ethnomedicinal Uses, Phytochemistry and Pharmacology of Alpinia Officinarum Hance. J. Ethnopharmacol. 224, 45–62. 10.1016/j.jep.2018.05.027 29803568

[B2] Aebi H. (1974). “Catalase,” in Editor Bergmeyer H. U. Methods of Enzymatic Analysis (New York: Elsevier), 673–684. 10.1016/b978-0-12-091302-2.50032-3

[B3] Al-Saikhan F. I. Ansari M. N. (2016). Evaluation of the Diuretic and Urinary Electrolyte Effects of Methanolic Extract of Peganum Harmala L. In Wistar Albino Rats. Saudi J. Biol. Sci. 23, 749–753. 10.1016/j.sjbs.2016.01.025 27872572 PMC5109494

[B4] Alvarez B. Demicheli V. Durán R. Trujillo M. Cerveñansky C. Freeman B. A. (2004). Inactivation of Human Cu,Zn Superoxide Dismutase by Peroxynitrite and Formation of Histidinyl Radical. Free Radic. Biol. Med. 37, 813–822. 10.1016/j.freeradbiomed.2004.06.006 15304256

[B5] Annuzzi G. Bozzetto L. Costabile G. Giacco R. Mangione A. Anniballi G. (2014). Diets Naturally Rich in Polyphenols Improve Fasting and Postprandial Dyslipidemia and Reduce Oxidative Stress: a Randomized Controlled Trial. Am. J. Clin. Nutr. 99, 463–471. 10.3945/ajcn.113.073445 24368433

[B6] Ansari M. N. (2015). Influence of Dietary Rocket Leaves on Diuresis and Urinary Electrolytes Excretion in High Fat Diet-Induced Obese Rats. Bull. Env. Pharmacol. Life Sci. 4, 9–13.

[B7] Ayele Y. Urga K. Engidawork E. (2010). Evaluation of *In Vivo* Antihypertensive and *In Vitro* Vasodepressor Activities of the Leaf Extract of Syzygium Guineense (Willd) D.C. Phytother. Res. 24, 1457–1462. 10.1002/ptr.3141 20878694

[B8] Basri A. M. Taha H. Ahmad N. (2017). A Review on the Pharmacological Activities and Phytochemicals of Alpinia Officinarum (Galangal) Extracts Derived from Bioassay-Guided Fractionation and Isolation. Pharmacogn. Rev. 11, 43–56. 10.4103/phrev.phrev_55_16 28503054 PMC5414456

[B9] Cavagni J. Wagner T. P. Gaio E. J. Rêgo R. O. C. C. Torres I. L. d. S. Rösing C. K. (2013). Obesity May Increase the Occurrence of Spontaneous Periodontal Disease in Wistar Rats. Arch. Oral Biol. 58, 1034–1039. 10.1016/j.archoralbio.2013.03.006 23562524

[B10] Chen F. Xiong H. Wang J. Ding X. Shu G. Mei Z. (2013). Antidiabetic Effect of Total Flavonoids from Sanguis Draxonis in Type 2 Diabetic Rats. J. Ethnopharmacol. 149, 729–736. 10.1016/j.jep.2013.07.035 23933499

[B11] Chen J. Zhao H. Ma X. Han X. Luo L. Wang L. (2012). The Effects of Jiang-Zhi-Ning and its Main Components on Cholesterol Metabolism. Evid-based Compl Alt. 2012. 10.1155/2012/928234 PMC3357595 22649479

[B12] Chen X.-Q. Hu T. Han Y. Huang W. Yuan H.-B. Zhang Y.-T. (2016). Preventive Effects of Catechins on Cardiovascular Disease. Molecules 21, 1759. 10.3390/molecules21121759 PMC6273873 28009849

[B13] Cho A.-S. Jeon S.-M. Kim M.-J. Yeo J. Seo K.-I. Choi M.-S. (2010). Chlorogenic Acid Exhibits Anti-obesity Property and Improves Lipid Metabolism in High-Fat Diet-Induced-Obese Mice. Food Chem. Toxicol. 48, 937–943. 10.1016/j.fct.2010.01.003 20064576

[B14] Dabeek W. M. Marra M. V. (2019). Dietary Quercetin and Kaempferol: Bioavailability and Potential Cardiovascular-Related Bioactivity in Humans. Nutrients 11, 2288. 10.3390/nu11102288 PMC6835347 31557798

[B15] de AF Da Silva R. d. C. de Souza P. Crestani S. Júnior A. G. Boligon A. A. Athayde M. L. (2015). Hypotensive and Diuretic Effect of the Butanolic Soluble Fraction of the Hydroethanolic Extract of Bark of Scutia Buxifolia Reissek in Rats. J. Ethnopharmacol. 172, 395–401. 10.1016/j.jep.2015.07.006 26164074

[B16] de Miranda A. M. Rossoni Júnior J. V. Souza e Silva L. Dos Santos R. C. Silva M. E. Pedrosa M. L. (2017). Agaricus Brasiliensis (Sun Mushroom) Affects the Expression of Genes Related to Cholesterol Homeostasis. Eur. J. Nutr*.* 56, 1707–1717. 10.1007/s00394-016-1217-x 27151383

[B17] Dobrian A. D. Davies M. J. Prewitt R. L. Lauterio T. J. (2000). Development of Hypertension in a Rat Model of Diet-Induced Obesity. Hypertension 35, 1009–1015. 10.1161/01.hyp.35.4.1009 10775577

[B18] Dobrian A. D. Schriver S. D. Lynch T. Prewitt R. L. (2003). Effect of Salt on Hypertension and Oxidative Stress in a Rat Model of Diet-Induced Obesity. Am. J. Physiol.-Renal Physiol. 285, F619–F628. 10.1152/ajprenal.00388.2002 12799306

[B19] Faulkner J. L. Belin de Chantemèle E. J. (2018). Sex Differences in Mechanisms of Hypertension Associated with Obesity. Hypertension 71, 15–21. 10.1161/hypertensionaha.117.09980 29133358 PMC5730468

[B20] Feng X. L. Pang M. Beard J. (2013). Health System Strengthening and Hypertension Awareness, Treatment and Control: Data from the China Health and Retirement Longitudinal Study. Bull. World Health Organ. 92, 29–41. 10.2471/blt.13.124495 24391298 PMC3865551

[B21] Férézou-Viala J. Roy A.-F. Sérougne C. Gripois D. Parquet M. Bailleux V. (2007). Long-term Consequences of Maternal High-Fat Feeding on Hypothalamic Leptin Sensitivity and Diet-Induced Obesity in the Offspring. Am. J. Physiology-Regulatory, Integr. Comp. Physiol. 293, R1056–R1062. 10.1152/ajpregu.00117.2007 17553843

[B22] Ferrell J. M. Pathak P. Boehme S. Gilliland T. Chiang J. Y. L. (2019). Deficiency of Both Farnesoid X Receptor and Takeda G Protein-Coupled Receptor 5 Exacerbated Liver Fibrosis in Mice. Hepatology 70, 955–970. 10.1002/hep.30513 30664797 PMC6642864

[B23] Garg A. Singh R. (2015). Antiobesity Activity of Aqueous and Ethanol Extracts of Aegle Marmelos Leaves in High Fat Diet Induced Obese Rats. Int. J. Pharm. Sci. Rev. Res. 30, 53–60.

[B24] Hailu W. Engidawork E. (2014). Evaluation of the Diuretic Activity of the Aqueous and 80% Methanol Extracts of Ajuga Remota Benth (Lamiaceae) Leaves in Mice. BMC Compl. Alternative. Med. 14, 135. 10.1186/1472-6882-14-135 PMC3997187 24720845

[B25] Harkness J. E. Turner P. V. VandeWoude S. Wheler C. L. (2010). Harkness and Wagner's Biology and Medicine of Rabbits and Rodents. Ames, IO: John Wiley & Sons.

[B26] Horáková Ľ. (2011). Flavonoids in Prevention of Diseases with Respect to Modulation of Ca-Pump Function. Interdiscip. Toxicol. 4, 114–124. 10.2478/v10102-011-0019-5 22058652 PMC3203913

[B27] Huang K. Huang Y. Frankel J. Addis C. Jaswani L. Wehner P. S. (2011). The Short-Term Consumption of a Moderately High-Fat Diet Alters Nitric Oxide Bioavailability in Lean Female Zucker Rats. Can. J. Physiol. Pharmacol. 89, 245–257. 10.1139/y11-016 21539468

[B28] Istvan E. S. (2002). Structural Mechanism for Statin Inhibition of 3-Hydroxy-3-Methylglutaryl Coenzyme A Reductase. Am. Heart J. 144, S27–S32. 10.1067/mhj.2002.130300 12486413

[B29] Kakkar P. Das B. Viswanathan P. (1984). A Modified Spectrophotometric Assay of Superoxide Dismutase. Indian J. Biochem. Biophys. 21, 130–132. 6490072

[B30] Kalaivani A. Uddandrao V. S. Parim B. Ganapathy S. Sushma N. Kancharla C. (2019). Reversal of High Fat Diet-Induced Obesity through Modulating Lipid Metabolic Enzymes and Inflammatory Markers Expressions in Rats. Arch. Physiol. Biochem. 125, 228–234. 10.1080/13813455.2018.1452036 29553847

[B31] Kelany M. E. Hakami T. M. Omar A. H. (2017). Curcumin Improves the Metabolic Syndrome in High-Fructose-Diet-Fed Rats: Role of TNF-α, NF-κB, and Oxidative Stress. Can. J. Physiol. Pharmacol. 95, 140–150. 10.1139/cjpp-2016-0152 27901349

[B32] Kim S. O. Yun S.-J. Lee E. H. (2007). The Water Extract of Adlay Seed (Coix Lachrymajobi Var. Mayuen) Exhibits Anti-obesity Effects through Neuroendocrine Modulation. Am. J. Chin. Med. 35, 297–308. 10.1142/s0192415x07004825 17436369

[B33] Kotsis V. Stabouli S. Papakatsika S. Rizos Z. Parati G. (2010). Mechanisms of Obesity-Induced Hypertension. Hypertens. Res. 33, 386–393. 10.1038/hr.2010.9 20442753

[B34] Lahlou S. Interaminense L. F. t. L. Leal-Cardoso J. H. Duarte G. P. (2003). Antihypertensive Effects of the Essential Oil of Alpinia Zerumbet and its Main Constituent, Terpinen-4-Ol, in DOCA-Salt Hypertensive Conscious Rats. Fund. Clin. Pharmacol. 17, 323–330. 10.1046/j.1472-8206.2003.00150.x 12803571

[B35] Lee M. O. (1929). Determination of the Surface Area of the white Rat with its Application to the Expression of Metabolic Results. Am. J. Physiol.-Legacy Content 89, 24–33. 10.1152/ajplegacy.1929.89.1.24

[B36] Li H. Matheny M. Nicolson M. Türner N. Scarpace P. J. (1997). Leptin Gene Expression Increases with Age Independent of Increasing Adiposity in Rats. Diabetes 46, 2035–2039. 10.2337/diab.46.12.2035 9392492

[B37] Liu Y. Huang C. Ceng C. Zhan H. Zheng D. Han W. (2014). Metformin Enhances Nitric Oxide Production and Diminishes Rho Kinase Activity in Rats with Hyperlipidemia. Lipids Health Dis. 13, 115. 10.1186/1476-511x-13-115 25028180 PMC4109376

[B38] Manosroi A. Lohcharoenkal W. Khonsung P. Manosroi W. Manosroi J. (2013). Potent Antihypertensive Activity of Thai-Lanna Medicinal Plants and Recipes from "MANOSROI III" Database. Pharm. Biol. 51, 1426–1434. 10.3109/13880209.2013.796391 23869399

[B39] Matsuda H. Nakashima S. Oda Y. Nakamura S. Yoshikawa M. (2009). Melanogenesis Inhibitors from the Rhizomes of Alpinia Officinarum in B16 Melanoma Cells. Bioorg. Med. Chem. 17, 6048–6053. 10.1016/j.bmc.2009.06.057 19615910

[B40] Modan M. Almog S. Fuchs Z. Chetrit A. Lusky A. Halkin H. (1991). Obesity, Glucose Intolerance, Hyperinsulinemia, and Response to Antihypertensive Drugs. Hypertension 17, 565–573. 10.1161/01.hyp.17.4.565 2013483

[B41] Nagao K. Inoue N. Wang Y.-M. Yanagita T. (2003). Conjugated Linoleic Acid Enhances Plasma Adiponectin Level and Alleviates Hyperinsulinemia and Hypertension in Zucker Diabetic Fatty (Fa/fa) Rats. Biochem. Biophys. Res. Commun. 310, 562–566. 10.1016/j.bbrc.2003.09.044 14521947

[B42] Neter J. E. Stam B. E. Kok F. J. Grobbee D. E. Geleijnse J. M. (2003). Influence of Weight Reduction on Blood Pressure. Hypertension 42, 878–884. 10.1161/01.hyp.0000094221.86888.ae 12975389

[B43] Nilvises N. Vamnatjinda V. Vanveerakul B. Pidech P. (1989). Diuretic Effect of Pluchea Indica. Thai. J. Pharmacol. 11, 1–8.

[B44] Ouchi N. Ohishi M. Kihara S. Funahashi T. Nakamura T. Nagaretani H. (2003). Association of Hypoadiponectinemia with Impaired Vasoreactivity. Hypertension 42, 231–234. 10.1161/01.hyp.0000083488.67550.b8 12860835

[B45] Panchal S. K. Brown L. (2010). Rodent Models for Metabolic Syndrome Research. J. Biomed. Biotechnol. 2011, 351982. 10.1155/2011/351982 21253582 PMC3018657

[B46] Parik T. Allikmets K. Teesalu R. Zilmer M. (1996). Oxidative Stress and Hyperinsulinaemia in Essential Hypertension: Different Facets of Increased Risk. J. Hypertens. 14, 407–410. 10.1097/00004872-199603000-00019 8723996

[B47] Ragab S. M. Elghaffar S. K. A. El-Metwally T. H. Badr G. Mahmoud M. H. Omar H. M. (2015). Effect of a High Fat, High Sucrose Diet on the Promotion of Non-alcoholic Fatty Liver Disease in Male Rats: the Ameliorative Role of Three Natural Compounds. Lipid Health Dis. 14, 83. 10.1186/s12944-015-0087-1 PMC4520282 26228038

[B48] Roberts C. K. Vaziri N. D. Wang X. Q. Barnard R. J. (2000). Enhanced NO Inactivation and Hypertension Induced by a High-Fat, Refined-Carbohydrate Diet. Hypertension 36, 423–429. 10.1161/01.hyp.36.3.423 10988276

[B49] Saleem U. Ahmad B. Ahmad M. Hussain K. Bukhari N. I. (2014). Investigation of *In Vivo* Antioxidant Activity of Euphorbia Helioscopia Latex and Leaves Methanol Extract: a Target against Oxidative Stress Induced Toxicity. Asian Pac. J. Trop. Med. 7, S369–S375. 10.1016/s1995-7645(14)60260-1 25312152

[B50] Selamoglu Z. (2017). Polyphenolic Compounds in Human Health with Pharmacological Properties. J. Trad Med. Clin. Naturo 6 (04), e137. 10.4172/2573-4555.1000e138

[B51] Sharma N. Okere I. Duda M. Johnson J. Yuan C. Chandler M. (2007). High Fructose Diet Increases Mortality in Hypertensive Rats Compared to a Complex Carbohydrate or High Fat Diet. Am. J. Hypertens. 20, 403–409. 10.1016/j.amjhyper.2006.09.022 17386347

[B52] Somova L. I. Shode F. O. Ramnanan P. Nadar A. (2003). Antihypertensive, Antiatherosclerotic and Antioxidant Activity of Triterpenoids Isolated from Olea Europaea, Subspecies Africana Leaves. J. Ethnopharmacol. 84, 299–305. 10.1016/s0378-8741(02)00332-x 12648829

[B53] Stec D. E. Gordon D. M. Hipp J. A. Hong S. Mitchell Z. L. Franco N. R. (2019). Loss of Hepatic PPARα Promotes Inflammation and Serum Hyperlipidemia in Diet-Induced Obesity. Am. J. Physiol.-Regulatory, Integr. Comp. Physiol. 317, R733–R745. 10.1152/ajpregu.00153.2019 PMC6879843 31483154

[B54] Tao L. Wang Z.-T. Zhu E.-Y. Lu Y.-H. Wei D.-Z. (2006). HPLC Analysis of Bioactive Flavonoids from the Rhizome of Alpinia Officinarum. South Afr. J. Bot. 72, 163–166. 10.1016/j.sajb.2005.06.007

[B55] Victório C. P. (2011). Therapeutic Value of the Genus Alpinia, Zingiberaceae. Rev. Bras. Farmacogn. 21, 194–201. 10.1590/s0102-695x2011005000025

[B56] Vogel H. G. Vogel W. H. (2013). Drug Discovery and Evaluation: Pharmacological Assays. New York: Springer Science & Business Media.

[B57] Wilson P. W. F. D'Agostino R. B. Sullivan L. Parise H. Kannel W. B. (2002). Overweight and Obesity as Determinants of Cardiovascular Risk. Arch. Intern. Med. 162, 1867–1872. 10.1001/archinte.162.16.1867 12196085

[B58] Xia D.-Z. Yu X.-F. Wang H.-M. Ren Q.-Y. Chen B.-M. (2010). Anti-Obesity and Hypolipidemic Effects of Ethanolic Extract fromAlpinia officinarumHance (Zingiberaceae) in Rats Fed High-Fat Diet. J. Med. Food 13, 785–791. 10.1089/jmf.2009.1235 20482258

[B59] Xie W. Wang W. Su H. Xing D. Cai G. Du L. (2007). Hypolipidemic Mechanisms of Ananas Comosus L. Leaves in Mice: Different from Fibrates but Similar to Statins. J. Pharmacol. Sci. 103, 267–274. 10.1254/jphs.fp0061244 17380035

[B60] Yan Y. Wang J. Chaudhry M. A. Nie Y. Sun S. Carmon J. (2019). Metabolic Syndrome and Salt-Sensitive Hypertension in Polygenic Obese TALLYHO/JngJ Mice: Role of Na/K-ATPase Signaling. Ijms 20, 3495. 10.3390/ijms20143495 PMC6678942 31315267

[B61] Yang J.-Y. Lee S.-J. Park H.-W. Cha Y.-S. (2006). Effect of Genistein with Carnitine Administration on Lipid Parameters and Obesity in C57Bl/6J Mice Fed a High-Fat Diet. J. Med. Food 9, 459–467. 10.1089/jmf.2006.9.459 17201630

[B62] Yao X. Lin Z. Jiang C. Gao M. Wang Q. Yao N. (2015). Cyclocarya Paliurus Prevents High Fat Diet Induced Hyperlipidemia and Obesity in Sprague-Dawley Rats. Can. J. Physiol. Pharmacol. 93, 677–686. 10.1139/cjpp-2014-0477 26203820

[B63] Zhan C.-D. Sindhu R. K. Pang J. Ehdaie A. Vaziri N. D. (2004). Superoxide Dismutase, Catalase and Glutathione Peroxidase in the Spontaneously Hypertensive Rat Kidney. J. Hypertens. 22, 2025–2033. 10.1097/00004872-200410000-00027 15361776

[B64] Zhang J.-Q. Wang Y. Li H.-L. Wen Q. Yin H. Zeng N.-K. (2015). Simultaneous Quantification of Seventeen Bioactive Components in Rhizome and Aerial Parts of Alpinia Officinarum Hance Using LC-MS/MS. Anal. Methods 7, 4919–4926. 10.1039/c5ay00647c

[B65] Zhi S. Cheng Z. Z. Cheng L. Z. (2002). Experimental Study of Diet-Induced Obesity Animal Model [J]. Chin. Pharmacol. Bull. 4.

